# Fuzzy Fractional Differential Equation Involving the Fuzzy Conformable Derivative and the *α* − Semigroups

**DOI:** 10.1155/2024/9993669

**Published:** 2024-02-08

**Authors:** Fouad Ibrahim Abdou Amir, Aziz El Ghazouani, M'hamed El Omari, Said Melliani

**Affiliations:** Laboratory of Applied Mathematics and Scientific Computing, Sultan Moulay Slimane University, Beni Mellal, Morocco

## Abstract

In this work, we discuss solutions to the abstract Cauchy problem of fuzzy conformal fractional. In addition, the method of fuzzy fractional semigroups is used to obtain analytical solutions to the fractional differential equation. We use the concept of Krasnoselskii's fixed point theorem to determine the existence and uniqueness of the solution. An application is also given to illustrate our main abstract results.

## 1. Introduction

Fuzzy fractional differential equations (FFDEs) have proved to be one of the most effective tools for modelling many phenomena in various fields of physics, mechanics, chemistry, and engineering. They have a large number of applications in the nonlinear oscillation of earthquakes, in many physical phenomena such as seepage flow in porous media, and in fluid dynamic circulation modelling. They have a large number of applications in the nonlinear oscillation of earthquakes, in many physical phenomena such as seepage flow in porous media, and in fluid dynamic circulation models. For more details on this theory and its applications, we refer to [1–7] [31–33].

Recently, Khalil et al. in [8] introduced a new differential operator, called the conformable derivative. This concept of conformal fractional calculus is a relatively new area of research which generalises the classical fractional calculus of noninteger degrees. This derivative of fractional order satisfies many well-known properties of the integer derivative, including linearity, the product rule, and the division rule. In addition, Rolle's theorem and the mean value theorem can also be applied, see [8]. One of the topics covered in this area concerns fractional semigroups, a type of operator semigroup whose generator is the conformable derivative. In 2015, Abdeljawad in [9] presented detailed results for the conformable derivative. In [10], Horani et al. describe fractional semigroups of operators and they further studied fractional abstract Cauchy problems with conformable derivatives introduced in [8]. Khalil et al. in [11] presented a geometric meaning of the conformable derivative through fractional sequences based on the conformable derivative. Abbeljawad et al. introduced the so-called *C*_0_-*α*-semigroup {*T*(*τ*)}_*τ*≥0_, which is a generalization of the classical strong continuous semigroup with its infinitesimal generator. Moreover, in [12], the authors introduced fuzzy fractional semigroups of operators associated with the congruent fuzzy fractional derivative. Numerous research papers using the congruent derivative have been published in recent years (see [13–27]).

The purpose of this work is to study the existence and uniqueness of fuzzy abstract Cauchy problems involving the fuzzy conformable derivatives using the method of fuzzy fractional semigroups.

We consider the following abstract fuzzy fractional conformal Cauchy problem:

(1) $$\begin{array}{c} \Psi^{\left(\alpha \right)}\left(\tau \right)=A\Psi \left(\tau \right)⊕\Phi \left(\tau ,\Psi \left(\tau \right)\right),\;\tau >0, \end{array}$$

(2) $$\begin{array}{c} \Psi \left(0\right)=\Psi_{0}, \end{array}$$

where Ψ^(*α*)^(*τ*) denotes the fuzzy conformable derivative, *A* : *𝒟*(*A*)⊆*𝒳*⟶*𝒳* is a linear operator with *𝒳*=*𝒞*([*a*, *b*], *ℝ*_*ℱ*_), and Φ(*τ*, Ψ(*τ*)) is continuously *α*− differentiable.

This paper is organized as follows: in Section 2, we provide all the necessary tools for fuzzy conformal fractional calculus and fuzzy fractional semigroups. In Section 3, we study the existence and uniqueness of the solution to problems (1) and (2). In Section 4, we give applications illustrating our abstract results. Finally, the article ends with a conclusion.

Let *𝒳*=*𝒞*([*a*, *b*], *ℝ*_*ℱ*_) be the space of continuous [*a*, *b*] valued functions in *ℝ*_*ℱ*_ and *ℒ*(*𝒳*, *𝒳*) be the space of all bounded operators on *𝒳*.

### 1.1. Notation

- *𝒳*=*𝒞*([*a*, *b*], *ℝ*_*ℱ*_): the space of continuous [*a*, *b*] valued functions in *ℝ*_*ℱ*_.
- *ℒ*(*𝒳*, *𝒳*): the space of all bounded operators on *𝒳*.
- *ℝ*_*ℱ*_: the space of fuzzy numbers.
- *𝒯*_*ϑ*_(*φ*)(*τ*) or *φ*^(*ϑ*)^(*τ*): fuzzy conformable derivative of *φ* of order *ϑ*.
- *ℑ*_*ϑ*_^*a*^(*φ*)(*τ*): conformable integral of *φ* of order *ϑ*.
-
0^ : the zero element on *ℝ*_*ℱ*_.
- ⊙: the multiplication on *ℝ*_*ℱ*_.
- ⊖: the subtraction on *ℝ*_*ℱ*_ also called *H*−difference.
- ⊖_*gh*_: generalize Hukuhara difference.
- ⊕: the addition on *ℝ*_*ℱ*_.
- {*T*(*τ*)}_*τ*≥0_: fuzzy *α*-semigroup.
- *A*: infinitesimal generator *a* of fuzzy *α*-semigroup.
- *𝒟*(*A*): domain of infinitesimal generator of a fuzzy *α*-semigroup.

## 2. Preliminaries

This section introduces the main properties of fuzzy number space and some other spaces based on it.

**Definition 1 .**

(see [28]). The space of fuzzy numbers denoted by *ℝ*_*ℱ*_ is defined as the class of fuzzy subsets of the real axis *ℝ*, i.e., of *u* : *ℝ*⟶[0,  1], having the following four properties:

- Normalization: sup_*x*∈*ℝ*_ *u*(*x*)=1.
- Convexity: for all *x*, *y* ∈ *ℝ* and all *λ* ∈ [0,  1], *u*(*λx*+(1 − *λ*)*y*) ≥ min(*u*(*x*), *u*(*y*)).
- Upper semicontinuity: for all *x* ∈ *ℝ*, the set {*y* ∈ *ℝ* : *u*(*y*) > *x*} is open.
- Compact support: the set {*x* ∈ *ℝ* : *u*(*x*) > 0} is bounded.

For all *r*∈ (0, 1], the *r*-level of an element of *ℝ*_*ℱ*_ is defined by

(3) $$\begin{array}{c} \omega^{r}=\left\{t\in R,\omega \left(t\right)\geq r\right\}. \end{array}$$

We can write in interval form as follows:

(4) $$\begin{array}{c} \omega^{r}=\left[\omega_{-}\left(r\right),\omega_{+}\left(r\right)\right]. \end{array}$$

(i) The addition of two fuzzy numbers is defined by ⊕:*ℝ*_*ℱ*_ × *ℝ*_*ℱ*_⟶*ℝ*_*ℱ*_,
  (5) $$\begin{array}{c} \left(\omega ⊕\upsilon \right)\left(x\right)=\underset{y+z=x}{\sup}\;\min\left\{\omega \left(y\right),\upsilon \left(z\right)\right\}. \end{array}$$
(ii) And multiplication by a scalar is defined by ⊙:*ℝ* × *ℝ*_*ℱ*_⟶*ℝ*_*ℱ*_,
  (6) $$\begin{array}{c} \left(\lambda ⊙\omega \right)\left(x\right)=\left\{\begin{array}{cc} \omega \left(\frac{x}{\lambda }\right), & \text{if}\;\lambda \neq 0,\\ \overset{˜}{0}, & \text{if}\;\lambda =0. \end{array}\right. \end{array}$$
Under *r*-level, we get for all *r* ∈ [0,  1],
  (7) $$\begin{array}{c} {\left[\omega ⊕\upsilon \right]}^{r}={\left[\omega \right]}^{r}+{\left[\upsilon \right]}^{r},\\ {\left[\lambda ⊙\upsilon \right]}^{r}=\lambda {\left[\upsilon \right]}^{r}. \end{array}$$
(iii) The subtraction in *ℝ*_*ℱ*_ is called the *H*-difference and is noted ⊖ defined as follows:
*ω* ⊖ *ϑ* makes sense if it exists *ξ* ∈ *ℝ*_*ℱ*_ such that *ω*=*ϑ* ⊕ *ξ*, for all *ω*, *ϑ* ∈ *ℝ*_*ℱ*_.
Clearly, *ω* ⊖ *ϑ* does not always exist.

We also define the generalized Hukuhara (gH) difference noted by ⊖_*gH*_ of two fuzzy numbers as follows:

(8) $$\begin{array}{c} \omega {⊖}_{gH}\vartheta =\xi ⟺\left\{\begin{array}{cc} \left(i\right), & \omega =\vartheta +\xi \;\text{or},\\ \left(ii\right), & \vartheta =\omega +\left(-1\right)\xi . \end{array}\right. \end{array}$$

For all, *ω*, *ϑ* ∈ *ℝ*_*ℱ*_.

**Definition 2 .**

(see [28]). The distance between two elements of *ℝ*_*ℱ*_ is given by

(9) $$\begin{array}{c} d\left(\omega ,\upsilon \right)=\underset{r\in \left[0,1\right]}{\sup}\;\max\left\{\left|\omega_{-}\left(r\right)-\upsilon_{-}\left(r\right)\right|,\left|\omega_{+}\left(r\right)\upsilon_{+}\left(r\right)\right|\right\}, \end{array}$$

verifying the following properties, for all *ω*, *ξ*, *κ*, *ϑ* ∈ *ℝ*_*ℱ*_.

- *d*(*ω* ⊕ *κ*, *ξ* ⊕ *κ*)=*d*(*ω*, *υ*).
- *d*(*γ*⊙*ω*, *γ*⊙*ξ*)=|*γ*|*d*(*ω*, *υ*).
- *d*(*ω* ⊕ *ξ*, *κ* ⊕ *ϑ*) ≤ *d*(*ω*, *κ*)+*d*(*ξ*, *ϑ*).

In what follows, we introduce the following Hukuhara's derivative.

**Definition 3 .**

(see [29]). Let *τ*_0_ ∈ (*a*, *b*) and *δ* be *τ*_0_+*δ* ∈ (*a*, *b*), then the generalized Hukuhara derivative of a fuzzy value function *ψ* : (*a*, *b*)⟶*ℝ*_*ℱ*_ at *τ*_0_ is defined as

(10) $$\begin{array}{c} \lim_{\delta ⟶0}\left\|\frac{\psi \left(\tau_{0}+\delta \right){⊖}_{gH}\psi \left(\tau_{0}\right)}{\delta }{⊖}_{gH}\psi_{gH}^{\prime }\left(\tau_{0}\right)\right\|=0. \end{array}$$

If *ψ*_*gH*_′(*τ*_0_) ∈ *ℝ*_*ℱ*_ satisfying (10) exists, we say that *ψ* is generalized Hukuhara differentiable (gH-differentiable for short) at *τ*_0_.

**Definition 4 .**

(see [29]). Let *ψ* : [*a*, *b*]⟶*ℝ*_*ℱ*_ and *τ*_0_ ∈ (*a*, *b*), with *ψ*_−_(*τ*, *r*) and *ψ*_+_(*τ*, *r*) both differentiable at *τ*_0_.

We say that

(1) *ψ* is [(*i*) − *gH*]-differentiable at *τ*_0_ if
  (11) $$\begin{array}{c} \psi_{i,gH}^{\prime }\left(\tau_{0}\right)=\left[\psi_{-}^{\prime }\left(\tau ,r\right),\psi_{+}^{\prime }\left(\tau ,r\right)\right]. \end{array}$$
(2) *ψ* is [(*ii*) − *gH*]-differentiable at *τ*_0_ if
  (12) $$\begin{array}{c} \psi_{ii,gH}^{\prime }\left(\tau_{0}\right)=\left[\psi_{+}^{\prime }\left(\tau ,r\right),\psi_{-}^{\prime }\left(\tau ,r\right)\right]. \end{array}$$

**Remark 5 .**

Clearly, Hukuhara differentiability implies generalized differentiability but not vice versa.

### 2.1. Conformable Derivative and Fuzzy *α*−Semigroup of Operator

The conformable derivative is a relatively new concept in fractional calculus. It is based on the basic limit definition of the derivative and differs from other fractional derivatives, such as the Riemann fractional derivative, in some key properties [9]. The conformable derivative has been studied in various contexts, including ordinary differential equations, complex-valued functions, and commutative algebras [9, 30].

The conformable derivative has been extended to arbitrary time scales, leading to the development of a new kind of conformable derivative on arbitrary time scales [5]. It has also been defined in finite-dimensional commutative associative algebras, demonstrating its applicability in various mathematical structures [6].

Here are some key properties and definitions of the conformable derivative:

**Definition 6 .**

(see [8]). Let *φ* ∈ *𝒞*([*a*, *b*], *ℝ*_*ℱ*_) and *ϑ* ∈ (0, 1).

Assuming *φ*(*τ*+*ε*(*τ* − *a*)^1−*ϑ*^)⊖_*gh*_*φ*(*τ*) exists, then we define the fuzzy conformable derivative of *φ* of order *ϑ* from

(13) $$\begin{array}{c} T_{\vartheta }\left(\varphi \right)\left(\tau \right)=\lim_{\varepsilon ⟶0}\frac{1}{\varepsilon }⊙\left(\varphi \left(\tau +\varepsilon {\left(\tau -a\right)}^{1-\vartheta }\right){⊖}_{gh}\varphi \left(\tau \right)\right), \end{array}$$

where *𝒯*_*ϑ*_(*φ*)(*τ*) can also be represented by *φ*^(*ϑ*)^(*τ*). If the fuzzy conformable derivative of *φ* of order *ϑ* exists, then we simply say *φ* is *ϑ*_*gh*_-differentiable

**Theorem 7 .**

If *φ*, a fuzzy function *φ*: [*a*, *∞*)⟶*ℝ*_*ℱ*_, is *ϑ*_*gh*_-differentiable at *τ*_0_ > 0, *ϑ* ∈ (0,  1), then *φ* is a fuzzy continuous function at *τ*_0_.

**Proof**

Indeed, we have

(14) $$\begin{array}{c} \varphi \left(\tau_{0}+\varepsilon \tau_{0}^{1-\vartheta }\right){⊖}_{gH}\varphi \left(\tau_{0}\right)\\ =\frac{\varphi \left(\tau_{0}+\varepsilon \tau_{0}^{1-\vartheta }\right){⊖}_{gH}\varphi \left(\tau_{0}\right)}{\varepsilon }⊙\varepsilon . \end{array}$$

Passing the limit, we get

(15) $$\begin{array}{c} \lim_{\varepsilon ⟶0}\left[\varphi \left(\tau_{0}+\varepsilon \tau_{0}^{1-\vartheta }\right){⊖}_{gH}\varphi \left(\tau_{0}\right)\right]\\ =\lim_{\varepsilon ⟶0}\frac{\varphi \left(\tau_{0}+\varepsilon \tau_{0}^{1-\vartheta }\right){⊖}_{gH}\varphi \left(\tau_{0}\right)}{\varepsilon }⊙\lim_{\varepsilon ⟶0}\varepsilon , \end{array}$$

then

(16) $$\begin{array}{c} \lim_{\varepsilon ⟶0}\left[\varphi \left(\tau_{0}+\varepsilon \tau_{0}^{1-\vartheta }\right){⊖}_{gH}\varphi \left(\tau_{0}\right)\right]=T_{\vartheta }\left(\varphi \right)\left(\tau_{0}\right)⊙0. \end{array}$$

Now, let *δ*=*ετ*_0_^1−*ϑ*^, therefore

(17) $$\begin{array}{c} \lim_{\delta ⟶0}\left[\varphi \left(\tau_{0}+\delta \right){⊖}_{gH}\varphi \left(\tau_{0}\right)\right]=0. \end{array}$$

Therefore, the function *φ* is a fuzzy continuous function.

**Theorem 8 .**

Let *ϑ* ∈ (0,  1) and *φ*: [*a*, *∞*) ⟶*ℝ*_*ℱ*_ be *ϑ*_*gh*_-differentiable fuzzy function at a point *τ* > 0. Then,

(18) $$\begin{array}{c} T_{\vartheta }\left(\varphi \right)\left(\tau \right)={\left(\tau -a\right)}^{1-\vartheta }⊙\varphi_{gh}^{\prime }\left(\tau \right). \end{array}$$

**Proof**

In Definition 6, let *δ*=*ε*(*τ* − *a*)^1−*ϑ*^ and then *ε*=(*τ* − *a*)^*ϑ*−1^*δ*. Hence,

(19) $$\begin{array}{c} T_{\vartheta }\left(\varphi \right)\left(\tau \right)=\lim_{\varepsilon ⟶0}\frac{\varphi \left(\tau +\varepsilon {\left(\tau -a\right)}^{1-\vartheta }\right){⊖}_{gH}\varphi \left(\tau \right)}{\varepsilon }\\ =\lim_{\delta ⟶0}\frac{\varphi \left(\tau +\delta \right){⊖}_{gH}\varphi \left(\tau \right)}{\delta {\left(\tau -a\right)}^{\vartheta -1}}\\ ={\left(\tau -a\right)}^{1-\vartheta }\lim_{\delta ⟶0}\frac{\varphi \left(\tau +\delta \right){⊖}_{gH}\varphi \left(\tau \right)}{\delta }\\ ={\left(\tau -a\right)}^{1-\vartheta }\varphi_{gH}^{\prime }\left(\tau \right). \end{array}$$

End of proof.

**Remark 9 .**

- The conformable derivative of a constant function is zero, which is not the case for Riemann fractional derivatives [9].
- The fuzzy conformable derivation satisfies all the classical properties of the derivation.

**Definition 10 .**

(see [7]). Let *φ* ∈ *𝒞*([*a*, *b*], *ℝ*_*ℱ*_) and *ϑ* ∈ (0,  1). The conformable integral is the inverse operation of the conformable derivative. It is defined as

(20) $$\begin{array}{c} I_{\vartheta }^{a}\left(\varphi \right)\left(\tau \right)=I_{1}^{a}\left({\left(\tau -a\right)}^{\vartheta -1}⊙\varphi \right)\\ =\int_{a}^{\tau }{\left(x-a\right)}^{\vartheta -1}⊙\varphi \left(x\right)dx. \end{array}$$

**Theorem 11 .**

Let *φ* be a fuzzy continuous function on [*a*, *b*], then the following properties are checked, for all *ϑ* ∈ (0,  1):

1. *𝒯*_*ϑ*_*ℑ*_*ϑ*_^*a*^(*φ*)(*τ*)=*φ*(*τ*).
2. *ℑ*_*ϑ*_^*a*^*𝒯*_*ϑ*_(*φ*)(*τ*)=*φ*(*τ*)⊖_*gH*_*φ*(*a*).

**Proof**

(1) Since *φ* is continuous, then *ℑ*_*ϑ*_^*a*^(*φ*)(*τ*) is clearly differentiable. Hence,
  (21) $$\begin{array}{c} T_{\vartheta }I_{\vartheta }^{a}\left(\varphi \right)\left(\tau \right)={\left(\tau -a\right)}^{1-\vartheta }\frac{d}{d\tau }I_{\vartheta }^{a}\left(\varphi \right)\left(\tau \right)\\ ={\left(\tau -a\right)}^{1-\vartheta }\frac{d}{d\tau }\int_{a}^{\tau }\frac{\varphi \left(x\right)}{{\left(x-a\right)}^{1-\vartheta }}dx\\ ={\left(\tau -a\right)}^{1-\vartheta }\frac{\varphi \left(\tau \right)}{{\left(\tau -a\right)}^{1-\vartheta }}\\ =\varphi \left(\tau \right). \end{array}$$
(2) We have
  (22) $$\begin{array}{c} I_{\vartheta }^{a}T_{\vartheta }\left(\varphi \right)\left(\tau \right)=\int_{a}^{\tau }{\left(x-a\right)}^{\vartheta -1}T_{\vartheta }\varphi \left(x\right)dx\\ =\int_{a}^{\tau }\varphi_{gh}^{\prime }\left(x\right)dx\\ =\varphi \left(\tau \right){⊖}_{gH}\varphi \left(a\right). \end{array}$$

**Theorem 12 (see [8]) (mean value theorem for conformable differentiable functions).**

Let *a* > 0 and *φ* : [*a*, *b*]⟶*ℝ*_*ℱ*_ be a given function satisfying the following:

- *φ* is continuous on [*a*, *b*].
- *φ* is *ϑ*-differentiable for some *ϑ* ∈ (0,  1).

Then, we have *c* ∈ (*a*, *b*), such that

(23) $$\begin{array}{c} \varphi^{\left(\vartheta \right)}\left(c\right)=\frac{\varphi \left(b\right){⊖}_{gH}\varphi \left(a\right)}{\left(1/\vartheta \right)b^{\vartheta }-\left(1/\vartheta \right)a^{\vartheta }}. \end{array}$$

Below we give some basic definitions of *α*−semigroups.

Let *𝒳*=*𝒞*([*a*, *b*], *ℝ*_*ℱ*_) be the space of continuous [*a*, *b*] valued functions in *ℝ*_*ℱ*_ and *ℒ*(*𝒳*, *𝒳*) is the space of all bounded operators on *𝒳*.

**Definition 13 .**

(see [12]). Let *α*∈ (0, *a*] for any *a* > 0. We call a fuzzy *α*-semigroup any operator {*T*(*τ*)}_*τ*≥0_ in *ℒ*(*𝒳*, *𝒳*) that verifies the following:

(i) *T*(0)=*I*, the identity mapping on *ℝ*_*ℱ*_.
(ii) *T*(*s*+*τ*)^1/*α*^=*T*(*s*^1/*α*^)*T*(*τ*^1/*α*^) for all *s*, *τ*∈ [0, *∞*).
(iii) There exist two constants *M* > 0 and *ω* such that, for *τ* ≥ 0, *x*, *y* ∈ *ℝ*_*ℱ*_, we have
  (24) $$\begin{array}{c} d\left(T{\left(\tau \right)}^{1/\alpha }x,T{\left(\tau \right)}^{1/\alpha }y\right)\leq Me^{\omega \tau \left(1/\alpha \right)}d\left(x,y\right). \end{array}$$

In particular, if *M*=1 and *ω*=0, we say that {*T*(*τ*)}_*τ*≥0_ is a contraction fuzzy *α*-semigroup.

**Remark 14 .**

Clearly, if *α*=1, then fuzzy 1-semigroups are just the fuzzy semigroups.

**Example 1 .**

(see [12]). Let *φ* ∈ *𝒞* ([0, +*∞*), *ℝ*_*ℱ*_), define the linear operator *T*(*τ*) by

(25) $$\begin{array}{c} \left(T\left(\tau \right)\varphi \right)\left(s\right)=\varphi \left(s+2\sqrt{\tau }\right), \end{array}$$

and {*T*(*τ*), *τ* ≥ 0} is a fuzzy 1/2−semigroup.

**Definition 15 .**

(see [12]). A fuzzy *α*-semigroup *T*(*τ*) is called a fuzzy *c*_0_-*α*-semigroup if, for each fixed *x* ∈ *𝒳*, *T*(*τ*)*x*⟶*x* as *τ*⟶0^+^.

We call the fuzzy conformable derivative of *T*(*τ*) at *τ*=0 the infinitesimal generator alpha of the fuzzy *α*−semigroup *T*(*τ*). It is denoted *A* and its domain is given by

(26) $$\begin{array}{c} D\left(A\right)=\left\{x\in X:\lim_{\tau ⟶0^{+}}T^{\left(\alpha \right)}\left(\tau \right)x\;\text{exists}\right\},\\ Ax=\lim_{\tau ⟶0^{+}}T^{\left(\alpha \right)}\left(\tau \right)x. \end{array}$$

**Theorem 16 .**

Given {*T*(*τ*)}_*τ*≥0_ a fuzzy *c*_0_-*α*-semigroup whose infinitesimal generator *A*, if *T*(*τ*) is continuously *α*-differentiable and *x* ∈ *𝒟*(*A*), then

(27) $$\begin{array}{c} T^{\left(\alpha \right)}\left(\tau \right)x=\text{AT}\left(\tau \right)x=T\left(\tau \right)Ax. \end{array}$$

**Proof**

Let us start with

(28) $$\begin{array}{c} T^{\left(\alpha \right)}\left(\tau \right)x\\ =\lim_{\varepsilon ⟶0}\frac{1}{\varepsilon }⊙\left(T\left(\tau +\varepsilon \tau^{1-\alpha }\right)x{⊖}_{gh}T\left(\tau \right)x\right)\\ =\lim_{\varepsilon ⟶0}\frac{1}{\varepsilon }⊙\left(T{\left(\tau^{\alpha }+{\left(\tau +\varepsilon \tau^{1-\alpha }\right)}^{\alpha }-\tau^{\alpha }\right)}^{1/\alpha }x{⊖}_{gh}T\left(\tau \right)x\right)\\ =\lim_{\varepsilon ⟶0}\frac{1}{\varepsilon }⊙\left(T{\left(\tau^{\alpha }+\left({\left(\tau +\varepsilon \tau^{1-\alpha }\right)}^{\alpha }-\tau^{\alpha }\right)\right)}^{1/\alpha }x{⊖}_{gh}T\left(\tau \right)x\right). \end{array}$$

As *T*(*τ*) is a fuzzy *α*-semigroup of operators, then

(29) $$\begin{array}{c} T{\left(a+b\right)}^{1/\alpha }=T\left(a^{1/\alpha }\right)T\left(b^{1/\alpha }\right). \end{array}$$

Thus,

(30) $$\begin{array}{c} T^{\left(\alpha \right)}\left(\tau \right)x\\ =\lim_{\varepsilon ⟶0}\frac{1}{\varepsilon }⊙\left(T{\left(\tau^{\alpha }\right)}^{1/\alpha }T{\left({\left(\tau +\varepsilon \tau^{1-\alpha }\right)}^{\alpha }-\tau^{\alpha }\right)}^{1/\alpha }x{⊖}_{gh}T\left(\tau \right)x\right)\\ =\lim_{\varepsilon ⟶0}\frac{1}{\varepsilon }⊙\left(T\left(\tau \right)\left[T{\left({\left(\tau +\varepsilon \tau^{1-\alpha }\right)}^{\alpha }-\tau^{\alpha }\right)}^{1/\alpha }x{⊖}_{gh}T\left(0\right)x\right]\right). \end{array}$$

If we now use the mean theorem for conformable derivatives defined in Theorem 12, we get

(31) $$\begin{array}{c} \frac{1}{\varepsilon }⊙\left(T\left(\tau \right)\left[T{\left({\left(\tau +\varepsilon \tau^{1-\alpha }\right)}^{\alpha }-\tau^{\alpha }\right)}^{1/\alpha }x{⊖}_{gh}T\left(0\right)x\right]\right)\\ =T\left(\tau \right)⊙T^{\left(\alpha \right)}\left(c\right)x\frac{\left[{\left(\tau +\varepsilon \tau^{1-\alpha }\right)}^{\alpha }-\tau^{\alpha }\right]}{\alpha \varepsilon }, \end{array}$$

for a few 0 < *c* < (*τ*+*ετ*^1−*α*^)^*α*^ − *τ*^*α*^.

If *ε*⟶0, then *c*⟶0, and lim_*ε*⟶0_ *T*^(*α*)^(*c*)=*T*^(*α*)^(0)=*A*. Hence,

(32) $$\begin{array}{c} T^{\left(\alpha \right)}\left(\tau \right)x=T\left(\tau \right)Ax\underset{\varepsilon ⟶0}{\;\lim}\frac{\left[{\left(\tau +\varepsilon \tau^{1-\alpha }\right)}^{\alpha }-\tau^{\alpha }\right]}{\alpha \varepsilon }. \end{array}$$

From L'Hopital's rule, we obtain

(33) $$\begin{array}{c} \lim_{\varepsilon ⟶0}\frac{\left[{\left(\tau +\varepsilon \tau^{1-\alpha }\right)}^{\alpha }-\tau^{\alpha }\right]}{\alpha \varepsilon }=1. \end{array}$$

So,

(34) $$\begin{array}{c} T^{\left(\alpha \right)}\left(\tau \right)x=T\left(\tau \right)Ax. \end{array}$$

Likewise, it can be shown that *T*(*τ*)*x* ∈ *𝒟*(*A*) and *T*^(*α*)^(*τ*)*x*=*AT*(*τ*)*x*.

In the following, we define *T* : [*a*, *b*]⟶*ℒ*(*𝒳*, *𝒳*) by

(35) $$\begin{array}{c} T\left(\tau \right)=\exp\left(\frac{1}{\alpha }\tau^{\alpha }A\right). \end{array}$$

It is clear that *T* is well a fuzzy *α*− semigroup.

Indeed,

(36) $$\begin{array}{c} T\left(0\right)=\exp\left(\frac{1}{\alpha }0^{\alpha }A\right)=I,\\ T{\left(\tau +s\right)}^{1/\alpha }=\exp\left(\frac{1}{\alpha }{\left[{\left(\tau +s\right)}^{1/\alpha }\right]}^{\alpha }A\right)\\ =\exp\left(\frac{1}{\alpha }\left(\tau +s\right)A\right)\\ =\exp\left(\frac{1}{\alpha }\tau A\right)\exp\left(\frac{1}{\alpha }sA\right)\\ =T{\left(\tau \right)}^{1/\alpha }T{\left(s\right)}^{1/\alpha }. \end{array}$$

what needed to be shown.

**Theorem 17 (Krasnoselskii's fixed point theorem).**

Let *M* be a closed convex and nonempty subset of a Banach space *X*. Let *A*, *B* be two operators such that

- *Ax*+*By* ∈ *M* whenever *x*, *y* ∈ *M*,
- *A* is compact and continuous,
- *B* is a contraction mapping.

Then, there exists *z* ∈ *M* such that *z*=*Az*+*Bz*.

## 3. Main Results

In this section, we show how our theory can be applied to provide solutions to specific problems. In particular, we would like to use the fractional semigroup concept to solve fuzzy differential problems.

### 3.1. The Fuzzy Differential Problem

We consider the following abstract fuzzy fractional conformal Cauchy problem:

(37) $$\begin{array}{c} \Psi^{\left(\alpha \right)}\left(\tau \right)=A\Psi \left(\tau \right)⊕\Phi \left(\tau ,\Psi \left(\tau \right)\right),\;\tau >0, \end{array}$$

(38) $$\begin{array}{c} \Psi \left(0\right)=\Psi_{0}, \end{array}$$

where Ψ^(*α*)^(*τ*) denotes the conformable derivative, *A* : *𝒟*(*A*)⊆*𝒳*⟶*𝒳* is a linear operator, and Φ(*τ*, Ψ(*τ*)) is continuously *α*_*gh*_− differentiable.

**Definition 18 .**

A function Ψ : [0, *a*]⟶*ℝ*_*ℱ*_ is a solution of the problems (37) and (38) if Ψ is continuous, is continuously *α*-differentiable on *ℝ*_*ℱ*_, belongs in *𝒟*(*A*), and satisfies (37) and (38).

**Theorem 19 .**

Let *𝒳* be a Banach space and *A* the infinitesimal generator of a fuzzy *c*_0_ − *α*− semigroup {*T*(*τ*)}_*τ*≥0_⊆*ℒ*(*𝒳*, *𝒳*). If Ψ_0_ ∈ *𝒟*(*A*) and Φ : ([*a*, *b*], *𝒳*)⟶*ℝ*_*ℱ*_ is continuous on ([*a*, *b*], *𝒳*), then problems (37) and (38) have a solution Ψ such that

(39) $$\begin{array}{c} \Psi \left(\tau \right)=T\left(\tau \right)\left(\Psi_{0}\right)⊕\int_{0}^{\tau }T{\left(\tau^{\alpha }-s^{\alpha }\right)}^{1/\alpha }\Phi \left(s,\Psi \left(s\right)\right)\text{ds}. \end{array}$$

**Proof**

For *τ* ≥ 0, we have

(40) $$\begin{array}{c} \Psi^{\left(\alpha \right)}\left(\tau \right)={\left[T\left(\tau \right)\left(\Psi_{0}\right)\right]}^{\left(\alpha \right)}\\ ⊕{\left(\int_{0}^{\tau }T{\left(\tau^{\alpha }-s^{\alpha }\right)}^{1/\alpha }\Phi \left(s,\Psi \left(s\right)\right)\text{ds}\right)}^{\left(\alpha \right)}. \end{array}$$

It is clear that [*T*(*τ*)(Ψ_0_)]^(*α*)^=*A*[*T*(*τ*)(Ψ_0_)]. We set

(41) $$\begin{array}{c} \Lambda \left(\tau \right)=\int_{0}^{\tau }T{\left(\tau^{\alpha }-s^{\alpha }\right)}^{1/\alpha }\Phi \left(s,\Psi \left(s\right)\right)\text{ds}. \end{array}$$

Let us prove that Λ^(*α*)^(*τ*) exists. Let *ε* > 0; we have

(42) $$\begin{array}{c} \Lambda \left(\tau +\varepsilon \tau^{1-\alpha }\right)\\ =\int_{0}^{\tau +\varepsilon \tau^{1-\alpha }}T{\left({\left(\tau +\varepsilon \tau^{1-\alpha }\right)}^{\alpha }-s^{\alpha }\right)}^{1/\alpha }\left(\Phi \left(s,\Psi \left(s\right)\right)\right)ds\\ =\int_{0}^{\tau +\varepsilon \tau^{1-\alpha }}T{\left({\left(\tau +\varepsilon \tau^{1-\alpha }\right)}^{\alpha }-\tau^{\alpha }+\tau^{\alpha }-s^{\alpha }\right)}^{1/\alpha }\\ ⊙\left(\Phi \left(s,\Psi \left(s\right)\right)\right)\text{ds}\\ =T{\left({\left(\tau +\varepsilon \tau^{1-\alpha }\right)}^{\alpha }-\tau^{\alpha }\right)}^{1/\alpha }\\ ⊙\left[\int_{0}^{\tau +\varepsilon \tau^{1-\alpha }}T{\left(\tau^{\alpha }-s^{\alpha }\right)}^{1/\alpha }\left(\Phi \left(s,\Psi \left(s\right)\right)\right)\text{ds}\right]\\ =T{\left({\left(\tau +\varepsilon \tau^{1-\alpha }\right)}^{\alpha }-\tau^{\alpha }\right)}^{1/\alpha }\\ ⊙\left[\Lambda \left(\tau \right)⊕\int_{\tau }^{\tau +\varepsilon \tau^{1-\alpha }}T{\left(\tau^{\alpha }-s^{\alpha }\right)}^{1/\alpha }\left(\Phi \left(s,\Psi \left(s\right)\right)\right)\text{ds}\right]\\ =T{\left({\left(\tau +\varepsilon \tau^{1-\alpha }\right)}^{\alpha }-\tau^{\alpha }\right)}^{1/\alpha }\left[\Lambda \left(\tau \right)\right]\\ ⊕T{\left({\left(\tau +\varepsilon \tau^{1-\alpha }\right)}^{\alpha }-\tau^{\alpha }\right)}^{1/\alpha }\\ ⊙\left[\int_{\tau }^{t+\varepsilon \tau^{1-\alpha }}T{\left(\tau^{\alpha }-s^{\alpha }\right)}^{1/\alpha }\left(\Phi \left(s,\Psi \left(s\right)\right)\right)\text{ds}\right]. \end{array}$$

But, there exists

(43) $$\begin{array}{c} T{\left({\left(\tau +\varepsilon \tau^{1-\alpha }\right)}^{\alpha }-\tau^{\alpha }\right)}^{1/\alpha }\left[\Lambda \left(\tau \right)\right]{⊖}_{gh}\Lambda \left(\tau \right), \end{array}$$

which implies

(44) $$\begin{array}{c} T{\left({\left(\tau +\varepsilon \tau^{1-\alpha }\right)}^{\alpha }-\tau^{\alpha }\right)}^{1/\alpha }\left[\Lambda \left(\tau \right)\right]\\ =\left[T{\left({\left(\tau +\varepsilon \tau^{1-\alpha }\right)}^{\alpha }-\tau^{\alpha }\right)}^{1/\alpha }\left(\Lambda \left(\tau \right)\right){⊖}_{gh}\Lambda \left(\tau \right)\right]\\ ⊕\Lambda \left(\tau \right). \end{array}$$

If we substitute the above and multiply by 1/*ε*, we get

(45) $$\begin{array}{c} \frac{1}{\varepsilon }⊙\left[\Lambda \left(\tau +\varepsilon \tau^{1-\alpha }\right){⊖}_{gh}\Lambda \left(\tau \right)\right]\\ =\frac{1}{\varepsilon }\left[T{\left({\left(\tau +\varepsilon t\tau^{1-\alpha }\right)}^{\alpha }-\tau^{\alpha }\right)}^{1/\alpha }\left(\Lambda \left(\tau \right)\right){⊖}_{gh}\Lambda \left(\tau \right)\right]\\ ⊕T{\left({\left(\tau +\varepsilon \tau^{1-\alpha }\right)}^{\alpha }-\tau^{\alpha }\right)}^{1/\alpha }\left[\frac{1}{\varepsilon }\right.\\ \left.⊙\int_{\tau }^{\tau +\varepsilon \tau^{1-\alpha }}T{\left(\tau^{\alpha }-s^{\alpha }\right)}^{1/\alpha }\left(\Phi \left(s,\Psi \left(s\right)\right)\right)\text{ds}\right]. \end{array}$$

When *ε*↘0, we get

(46) $$\begin{array}{c} \lim_{\varepsilon ⟶0}\frac{1}{\varepsilon }⊙\left[T{\left({\left(\tau +\varepsilon \tau^{1-\alpha }\right)}^{\alpha }-\tau^{\alpha }\right)}^{1/\alpha }\Lambda \left.\left(\tau \right)\right){⊖}_{gh}\Lambda \left(\tau \right)\right]\\ =\lim_{\varepsilon ⟶0}\frac{1}{\varepsilon }\left[T{\left({\left(\tau +\varepsilon \tau^{1-\alpha }\right)}^{\alpha }-\tau^{\alpha }\right)}^{1/\alpha }{⊖}_{gh}T\left(0\right)\right]\Lambda \left(\tau \right)\\ =\lim_{\varepsilon ⟶0}{\;T}^{\left(\alpha \right)}\left(c\right)\Lambda \left(\tau \right)\left[\frac{\left({\left(\tau +\varepsilon \tau^{1-\alpha }\right)}^{\alpha }-\tau^{\alpha }\right)}{\alpha \varepsilon }\right], \end{array}$$

with

(47) $$\begin{array}{c} 0<c<{\left(\tau +\varepsilon \tau^{1-\alpha }\right)}^{\alpha }-\tau^{\alpha }, \end{array}$$

thus, if *ε*⟶0, then *c*⟶0 and

(48) $$\begin{array}{c} \lim_{\varepsilon ⟶0}{\;T}^{\left(\alpha \right)}\left(c\right)=T^{\left(\alpha \right)}\left(0\right)=A. \end{array}$$

Hence,

(49) $$\begin{array}{c} \lim_{\varepsilon ⟶0}\;\frac{1}{\varepsilon }⊙\left[T{\left({\left(\tau +\varepsilon \tau^{1-\alpha }\right)}^{\alpha }-\tau^{\alpha }\right)}^{1/\alpha }\left(\Lambda \left(\tau \right)\right){⊖}_{gh}\Lambda \left(\tau \right)\right]\\ =A\left[\Lambda \left(\tau \right)\right]\lim_{\varepsilon ⟶0}\left[\frac{\left({\left(\tau +\varepsilon \tau^{1-\alpha }\right)}^{\alpha }-\tau^{\alpha }\right)}{\alpha \varepsilon }\right]\\ =A\left[\Lambda \left(\tau \right)\right]. \end{array}$$

This implies the following:

(50) $$\begin{array}{c} \lim_{\varepsilon ⟶0}\frac{1}{\varepsilon }⊙\left[\Lambda \left(\tau +\varepsilon \tau^{1-\alpha }\right){⊖}_{gh}\Lambda \left(\tau \right)\right]\\ =A\left[\Lambda \left(\tau \right)\right]⊕\lim_{\varepsilon ⟶0}\;T{\left({\left(\tau +\varepsilon \tau^{1-\alpha }\right)}^{\alpha }-\tau^{\alpha }\right)}^{1/\alpha }\\ ⊙\left[\frac{1}{\varepsilon }⊙\int_{\tau }^{\tau +\varepsilon \tau^{1-\alpha }}T{\left(\tau^{\alpha }-s^{\alpha }\right)}^{1/\alpha }\left(\Phi \left(s,\Psi \left(s\right)\right)\right)\text{ds}\right]\\ =A\left[\Lambda \left(\tau \right)\right]⊕T{\left(0\right)}^{\frac{1}{\alpha }}\left[\lim_{\varepsilon ⟶0}\frac{1}{\varepsilon }⊙\int_{\tau }^{\tau +\varepsilon \tau^{1-\alpha }}T{\left(\tau^{\alpha }-s^{\alpha }\right)}^{1/\alpha }\left(\Phi \left(s,\Psi \left(s\right)\right)\right)\text{ds}\right]\\ =A\left[\Lambda \left(\tau \right)\right]⊕\left[\lim_{\varepsilon ⟶0}\frac{1}{\varepsilon }⊙\int_{\tau }^{\tau +\varepsilon \tau^{1-\alpha }}T{\left(\tau^{\alpha }-s^{\alpha }\right)}^{1/\alpha }\left(\Phi \left(s,\Psi \left(s\right)\right)\right)\text{ds}\right]. \end{array}$$

This remains to be proven.

(51) $$\begin{array}{c} \lim_{\varepsilon ⟶0}\frac{1}{\varepsilon }⊙\int_{\tau }^{\tau +\varepsilon \tau^{1-\alpha }}T{\left(\tau^{\alpha }-s^{\alpha }\right)}^{1/\alpha }\left(\Phi \left(s,\Psi \left(s\right)\right)\right)\text{ds}\\ =\Phi \left(\tau ,\Psi \left(\tau \right)\right). \end{array}$$

Indeed, given that

(52) $$\begin{array}{c} \Phi \left(\tau ,\Psi \left(\tau \right)\right)=T\left(0\right)\Phi \left(\tau ,\Psi \left(\tau \right)\right)\\ =\frac{1}{\varepsilon \tau^{1-\alpha }}⊙\int_{\tau }^{\tau +\varepsilon \tau^{1-\alpha }}T\left(0\right)\left(\Phi \left(\tau ,\Psi \left(\tau \right)\right)\right)\text{ds}, \end{array}$$

we have

(53) $$\begin{array}{c} d\left(\frac{1}{\varepsilon }⊙\int_{\tau }^{\tau +\varepsilon \tau^{1-\alpha }}T{\left(\tau^{\alpha }-s^{\alpha }\right)}^{1/\alpha }\left(\Phi \right.\left(s,\Psi \left(\left(s\right)\right)\right)\text{ds},\frac{1}{\varepsilon \tau^{1-\alpha }}⊙\int_{\tau }^{\tau +\varepsilon \tau^{1-\alpha }}T\left(0\right)\left(\Phi \right.\left(\tau ,\Psi \left(\left(\tau \right)\right)\right)\text{ds}\right)\\ \leq d\left(\frac{1}{\varepsilon }⊙\int_{\tau }^{\tau +\varepsilon \tau^{1-\alpha }}T{\left(\tau^{\alpha }-s^{\alpha }\right)}^{1/\alpha }\left(\Phi \right.\left(s,\Psi \left(\left(s\right)\right)\right)\text{ds},\frac{1}{\varepsilon }⊙\int_{\tau }^{\tau +\varepsilon \tau^{1-\alpha }}T\left(0\right)\left(\Phi \right.\left(\tau ,\Psi \left(\left(\tau \right)\right)\right)\text{ds}\right)\\ \leq \frac{1}{\varepsilon }\int_{\tau }^{\tau +\varepsilon \tau^{1-\alpha }}H_{\tau }\left(s\right)\text{ds}, \end{array}$$

where

(54) $$\begin{array}{c} H_{\tau }\left(s\right)=d\left[T{\left(\tau^{\alpha }-s^{\alpha }\right)}^{1/\alpha }\left(\Phi \left(s,\Psi \right)\right),T\left(0\right)\left(\Phi \left(\tau ,\Psi \right)\right)\right], \end{array}$$

is continuous on [*τ*, *τ*+*ετ*^1−*α*^] as a function of *s* because *T*(·) and Φ are continuous. Therefore,

(55) $$\begin{array}{c} \frac{1}{\varepsilon }\int_{\tau }^{\tau +\varepsilon \tau^{1-\alpha }}H_{\tau }\left(s\right)\text{ds}⟶H_{\tau }\left(\tau \right)=0,\text{as}\;\varepsilon ↘0. \end{array}$$

Hence,

(56) $$\begin{array}{c} \lim_{\varepsilon ⟶0}\frac{1}{\varepsilon }⊙\left[\Lambda \left(\tau +\varepsilon \tau^{1-\alpha }\right){⊖}_{gh}\Lambda \left(\tau \right)\right]\\ =A\left[\Lambda \left(\tau \right)\right]⊕\Phi \left(\tau \right.,\Psi \left(\left(\tau \right)\right). \end{array}$$

To conclude, we have, for *τ* ≥ 0,

(57) $$\begin{array}{c} \Lambda^{\left(\alpha \right)}\left(\tau \right)=A\left[\Lambda \left(\tau \right)\right]⊕\Phi \left(\tau \right.,\Psi \left(\left(\tau \right)\right). \end{array}$$

Thus,

(58) $$\begin{array}{c} \Psi^{\left(\alpha \right)}\left(\tau \right)=A\left[T\left(\tau \right)\left(\Psi_{0}\right)\right]⊕A\left[\Lambda \left(\tau \right)\right]⊕\Phi \left(\tau \right.,\Psi \left(\left(\tau \right)\right)\\ =A\left[\Psi \left(\tau \right)\right]⊕\Phi \left(\tau \right.,\Psi \left(\left(\tau \right)\right). \end{array}$$

This completes the proof.

In the following, let us show the existence and uniqueness of the solution.

### 3.2. Existence and Uniqueness of the Solution

**Theorem 20 .**

Suppose a bounded continuous function *g* satisfies the following condition:

(59) $$\begin{array}{c} d\left(\Phi \left(\tau ,\Psi \right),\Phi \left(\tau ,\phi \right)\right)\leq \lambda d\left(\Psi ,\phi \right),\;\Psi ,\phi \in R_{F},\\ \lambda \in R\;\text{suchthat}\;\lambda <b^{\alpha -1}\left\|A\right\|. \end{array}$$

Then, using Krasnoselskii's fixed point theorem, equations (37) and (38) have at least one solution.

**Proof**

Consider a closed convex subset that is not empty

(60) Bk=Ψ∈Ca,b×RF,RF,dΨ,0^≤k,

where

(61) $$\begin{array}{c} k=M+Nb^{1-\alpha }{\left\|A\right\|}^{-1}, \end{array}$$

with

(62) $$\begin{array}{c} M=\left\|T\left(t\right)\left(u_{0}\right)\right\|,\\ N=\left\|\Phi \left(\tau ,\Psi \left(\tau \right)\right)\right\|. \end{array}$$

Define the operators *𝒯*_1_ and *𝒯*_2_ on *𝔅*_*k*_ by

(63) $$\begin{array}{c} T_{1}\Psi \left(\tau \right)=T\left(\tau \right)\left(\Psi_{0}\right),\\ T_{2}\Psi \left(\tau \right)=\int_{0}^{\tau }T{\left(\tau^{\alpha }-s^{\alpha }\right)}^{1/\alpha }\Phi \left(s,\Psi \left(s\right)\right)\text{ds}. \end{array}$$

Thus, Ψ being a fixed point of the operator *𝒯*Ψ=*𝒯*_1_Ψ+*𝒯*_2_Ψ is a solution of equations (37) and (38).

In the first step, we prove that *𝒯* maps *𝔅*_*k*_ into *𝔅*_*k*_, i.e., for any Ψ, *ϕ* ∈ *𝔅*_*k*_.

We have to show that *𝒯*_1_Ψ+*𝒯*_2_*ϕ* ∈ *𝔅*_*k*_:

(64) dT1Ψ+T2ϕ,0^=TτΨ0+∫0τTτα−sα1/αΦs,ϕsds≤TτΨ0+∫0τTτα−sα1/αΦs,ϕsds≤M+N∫0τTτα−sα1/αds≤M+N∫0τ exp1ατα−sαAds≤M+N exp1αταA∫0τ exp1α−sαAds≤M+N exp1αταA−s1−αA−1 exp1α−sαA0τ≤M+N exp1αταA−τ1−αA−1 exp1α−ταA≤M+Nb1−αA−1=k.

Therefore, *𝔅*_*k*_ ⊂ *𝔅*_*k*_. Now, to prove that *𝒯*_1_ is continuous, let us consider a sequence Ψ_*n*_ such that Ψ_*n*_⟶Ψ

(65) $$\begin{array}{c} d\left(T_{1}\Psi_{n}\left(\tau \right),T_{1}\Psi \left(\tau \right)\right)=d\left(T\left(\tau \right)\Psi_{0},T\left(\tau \right)\Psi_{0}\right)=0. \end{array}$$

Hence, *𝒯*_1_ is continuous. Now, we show that *𝒯*_1_(*𝔅*_*k*_) resides in a relatively compact set. Taking *τ*_1_ ≤ *τ*_2_ ≤ *T*, we have

(66) $$\begin{array}{c} d\left(T_{1}\Psi \left(\tau_{1}\right),T_{1}\Psi \left(\tau_{2}\right)\right)=d\left(T\left(\tau_{1}\right)\Psi_{0},T\left(\tau_{2}\right)\Psi_{0}\right),\\ =\left\|T\left(\tau_{1}\right)\Psi_{0}-T\left(\tau_{2}\right)\Psi_{0}\right\|. \end{array}$$

As *τ*_1_⟶*τ*_2_, we get *𝒯*_1_Ψ(*τ*_1_)⟶*𝒯*_1_Ψ(*τ*_2_). Hence, *𝒯*_1_(*𝔅*_*k*_) resides in a relatively compact. Now, we show that *𝒯*_2_ is contraction. Letting Ψ, *ϕ* ∈ *𝔅*_*k*_, we have

(67) $$\begin{array}{c} d\left(T_{2}\right.\Psi \left(\tau \right),T_{2}\phi \left(\tau \right)\\ =\left\|\int_{0}^{\tau }T{\left(\tau^{\alpha }-s^{\alpha }\right)}^{1/\alpha }\left(\Phi \left(s,\Psi \left(s\right)\right)-\Phi \left(s\right.,\phi \left(\left(s\right)\right)\right)\text{ds}\right\|\\ \leq \int_{0}^{\tau }T{\left(\tau^{\alpha }-s^{\alpha }\right)}^{1/\alpha }\left\|\Phi \left(s,\Psi \left(s\right)\right)-\Phi \left(s\right.,\phi \left(\left(s\right)\right)\right\|\text{ds}\\ \leq \lambda \left\|\Psi -\phi \right\|\int_{0}^{\tau }T{\left(\tau^{\alpha }-s^{\alpha }\right)}^{1/\alpha }\text{ds}\\ \leq b^{1-\alpha }{\left\|A\right\|}^{-1}\lambda \left\|\Psi -\phi \right\|. \end{array}$$

For *λ* ≤ ‖*A*‖*b*^*α*−1^, then *𝒯*_2_ is contraction.

Hence, according to Theorem 17, *𝒯* has a fixed point in *𝔅*_*k*_ which is a solution of equations (37) and (38).

## 4. Application to Fuzzy Differential Equation

In this section, we apply the main results of the previous sections to solve fuzzy fractional differential equations. We consider the following fuzzy fractional partial differential equation with initial conditions.

(68) $$\begin{array}{c} \left\{\begin{array}{c} \psi^{\left(\alpha \right)}\left(\tau ,x\right)=\phi \left(\tau ,x\right)⊕\tau ⊙\sin\left(\psi \left(\tau ,x\right)\right),\\ \phi^{\left(\alpha \right)}\left(\tau ,x\right)=\psi \left(t,x\right)⊕\tau ⊙\sin\left(\phi \left(\tau ,x\right)\right),\tau \in \left[0,\;1\right], \end{array}\right.\\ \left\{\begin{array}{c} \psi \left(0,x\right)=\psi_{0}\left(x\right),\\ \phi \left(0,x\right)=\phi_{0}\left(x\right),x\in \left[a,b\right], \end{array}\right. \end{array}$$

where *ψ*^(*α*)^ means the fuzzy conformable derivative of *ψ* with respect to *τ*.

Let us put

(69) $$\begin{array}{c} A=\left(\begin{array}{cc} 0 & 1\\ 1 & 0 \end{array}\right),\Psi =\left(\begin{array}{c} \psi \\ \phi \end{array}\right),\Psi_{0}=\left(\begin{array}{c} \psi_{0}\\ \phi_{0} \end{array}\right),\\ \Phi \left(\tau ,\Psi \right)=\left(\begin{array}{c} \tau ⊙\sin\left(\psi \left(\tau ,x\right)\right)\\ \tau ⊙\sin\left(\phi \left(\tau ,x\right)\right) \end{array}\right). \end{array}$$

Then, problem (68) can be written as

(70) $$\begin{array}{c} \left\{\begin{array}{c} \Psi^{\left(\alpha \right)}\left(\tau \right)=\overset{˜}{A}\left[\Psi \left(t\right)\right]⊕\Phi \left(\tau ,\Psi \left(\tau \right)\right),\tau \geq 0,\\ \Psi \left(0\right)=\Psi_{0}, \end{array}\right. \end{array}$$

where

(71) $$\begin{array}{c} \overset{˜}{A}\in {\left[C\left(\left[a,b\right];R_{F}\right)\right]}^{2}⟶{\left[C\left(\left[a,b\right];R_{F}\right)\right]}^{2}, \end{array}$$

is defined by

(72) $$\begin{array}{c} \overset{˜}{A}\left(\Psi \right)=A⊙\Psi =\left(\begin{array}{c} \phi \\ \psi \end{array}\right). \end{array}$$

Clearly, *A* is a linear operator and continuous at each Ψ. Then, according to Theorem 19, the solution of (68) is written as

(73) $$\begin{array}{c} \Psi \left(\tau \right)=T\left(\tau \right)\left(\Psi_{0}\right)⊕\int_{0}^{\tau }T{\left(\tau^{\alpha }-s^{\alpha }\right)}^{1/\alpha }\Phi \left(s,\Psi \left(s\right)\right)\text{ds}, \end{array}$$

where

(74) $$\begin{array}{c} T\left(\tau \right)=\exp\left(\frac{1}{\alpha }\tau^{\alpha }⊙A\right). \end{array}$$

We will calculate exp(1/*ατ*^*α*^⊙*A*). In this case, we get that

(75) $$\begin{array}{c} A^{n}=\left\{\begin{array}{cc} I, & \text{if}\;n\;\text{is}\;\text{even},\\ A, & \text{if}\;n\;\text{is}\;\text{odd}. \end{array}\right. \end{array}$$

Thus,

(76) $$\begin{array}{c} T\left(\tau \right)\Psi_{0}=\exp\left(\frac{1}{\alpha }\tau^{\alpha }⊙A\right)⊙\Psi_{0}\\ =\overset{+\infty }{\underset{k=0}{\sum }}\frac{{\left(\left(1/\alpha \right)\tau^{\alpha }\right)}^{k}A^{k}}{k!}\Psi_{0}\\ =\Psi_{0}I+\frac{\left(1/\alpha \right)\tau^{\alpha }A}{1!}\Psi_{0}+\frac{{\left(\left(1/\alpha \right)\tau^{\alpha }\right)}^{2}I}{2!}\Psi_{0}\\ +\frac{{\left(\left(1/\alpha \right)\tau^{\alpha }\right)}^{3}A}{3!}\Psi_{0}+\frac{{\left(\left(1/\alpha \right)\tau^{\alpha }\right)}^{4}I}{4!}\Psi_{0}+\cdots \\ =\Psi_{0}\;\cosh\left(\frac{1}{\alpha }\tau^{\alpha }\right)+\sinh\left(\frac{1}{\alpha }\tau^{\alpha }\right)\overset{˜}{A}\left[\Psi_{0}\right]. \end{array}$$

Hence,

(77) $$\begin{array}{c} \Psi \left(\tau \right)=T\left(\tau \right)\left(\Psi_{0}\right)⊕\int_{0}^{t}\tau T{\left(\tau^{\alpha }-s^{\alpha }\right)}^{1/\alpha }\Phi \left(s,\Psi \left(s\right)\right)\text{ds}\\ =\Psi_{0}\;\cosh\left(\frac{1}{\alpha }\tau^{\alpha }\right)+\sinh\left(\frac{1}{\alpha }\tau^{\alpha }\right)\overset{˜}{A}\left[\Psi_{0}\right]\\ ⊕\int_{0}^{\tau }T{\left(\tau^{\alpha }-s^{\alpha }\right)}^{1/\alpha }\Phi \left(s,\Psi \left(s\right)\right)\text{ds}, \end{array}$$

i.e.,

(78) $$\begin{array}{c} \psi \left(\tau ,x\right)=\cosh\left(\frac{1}{\alpha }\tau^{\alpha }\right)\psi_{0}\left(x\right)⊕\sinh\left(\frac{1}{\alpha }\tau^{\alpha }\right)\phi_{0}\left(x\right)\\ ⊕\int_{0}^{\tau }\;\exp\left(\frac{1}{\alpha }\left(\tau^{\alpha }-s^{\alpha }\right)A\right)\tau ⊙\sin\left(\psi \left(\tau ,x\right)\right)\text{ds},\\ \phi \left(\tau ,x\right)=\sinh\left(\frac{1}{\alpha }\tau^{\alpha }\right)\psi_{0}\left(x\right)⊕\cosh\left(\frac{1}{\alpha }\tau^{\alpha }\right)\phi_{0}\left(x\right)\\ ⊕\int_{0}^{\tau }\;\exp\left(\frac{1}{\alpha }\left(\tau^{\alpha }-s^{\alpha }\right)A\right)\tau ⊙\sin\left(\phi \left(\tau ,x\right)\right)\text{ds}. \end{array}$$

As *τ*⊙sin(*ψ*(*τ*, *x*)) is Lipschitz with respect to *τ* and satisfies the hypothesis of Theorem 20, then *ψ*(*τ*, *x*) and *ϕ*(*τ*, *x*) are unique.

## 5. Conclusion

In this study, the initial problem of fuzzy conformal orders is discussed in the context of conformable generalized Hukuhara differentiability. Fuzzy conformable derivatives based on generalized Hukuhara differentiability are introduced, and many relevant properties of this topic are shown [27]. Therefore, the fuzzy semifractional group method is used to determine the analytical solution of the conformable fractional differential equation. To ensure the existence and uniqueness of the solution, we use Krasnoselskii's fixed point theorem. Finally, the application of abstract Cauchy problems is highlighted to demonstrate the effectiveness and efficiency of these methods. The results show that the conformable fuzzy fractional-order semigroup method is an effective and practical tool for solving conformable fuzzy fractional-order differential equations. We obtain interesting results here that can be used in future studies of fuzzy fractional partial differential equations under conformable gH differentiability.

## Data Availability

The data used to support the findings of this study are available from the corresponding author upon request.
